# Identification of the role of immune-related genes in the diagnosis of bipolar disorder with metabolic syndrome through machine learning and comprehensive bioinformatics analysis

**DOI:** 10.3389/fpsyt.2023.1187360

**Published:** 2023-10-04

**Authors:** Jing Shen, Yu Feng, Minyan Lu, Jin He, Huifeng Yang

**Affiliations:** ^1^The Affiliated Jiangsu Shengze Hospital of Nanjing Medical University, Nanjing, China; ^2^Medicine and Health, The University of New South Wales, Kensington, NSW, Australia; ^3^Melbourne Medical School, The University of Melbourne, Parkville, VIC, Australia

**Keywords:** bipolar disorder, metabolic syndrome, differentially expressed genes, machine learning, immune infiltration bipolar disorder, immune infiltration

## Abstract

**Background:**

Bipolar disorder and metabolic syndrome are both associated with the expression of immune disorders. The current study aims to find the effective diagnostic candidate genes for bipolar affective disorder with metabolic syndrome.

**Methods:**

A validation data set of bipolar disorder and metabolic syndrome was provided by the Gene Expression Omnibus (GEO) database. Differentially expressed genes (DEGs) were found utilizing the Limma package, followed by weighted gene co-expression network analysis (WGCNA). Further analyses were performed to identify the key immune-related center genes through function enrichment analysis, followed by machine learning-based techniques for the construction of protein–protein interaction (PPI) network and identification of the Least Absolute Shrinkage and Selection Operator (LASSO) and Random Forest (RF). The receiver operating characteristic (ROC) curve was plotted to diagnose bipolar affective disorder with metabolic syndrome. To investigate the immune cell imbalance in bipolar disorder, the infiltration of the immune cells was developed.

**Results:**

There were 2,289 DEGs in bipolar disorder, and 691 module genes in metabolic syndrome were identified. The DEGs of bipolar disorder and metabolic syndrome module genes crossed into 129 genes, so a total of 5 candidate genes were finally selected through machine learning. The ROC curve results-based assessment of the diagnostic value was done. These results suggest that these candidate genes have high diagnostic value.

**Conclusion:**

Potential candidate genes for bipolar disorder with metabolic syndrome were found in 5 candidate genes (AP1G2, C1orf54, DMAC2L, RABEPK and ZFAND5), all of which have diagnostic significance.

## 1. Introduction

Bipolar disorder is a serious mental illness characterized by depression, mania, and mixed development (1). Patients often have persistent residual symptoms, psychosocial function problems, cognitive impairment, and low quality of life, along with other complications (2). Bipolar disorder is often missed or misdiagnosed, which makes the disease statistics much lower than its actual prevalence (3). The disease has also been proven to be a chronic hereditary disease (4), and environmental factors have a significant influence on the development and recurrence of the condition (5). Therefore, genetic analysis of patients will provide more support for future diagnosis and therapy. According to prior research, there are multiple correlation patterns between bipolar disorder and various medical diseases involving multiple organ systems (6).

The term “metabolic syndrome” describes the co-existence of numerous recognized cardiovascular risk factors (obesity, insulin resistance, atherogenic dyslipidemia, and hypertension) (7). The disease has grown to be a significant health risk in the modern world as western habits have become more widespread (8). For example, the overall standardized prevalence rate of metabolic syndrome in China is 24.2% (9), which may be related to the high local drinking rate (the drinking rate of Chinese adults is 33.66%) (10), making it one of the major diseases in China.

The lifetime attempted suicide rate of bipolar disorder is 32.3% (11), and the premature mortality rate is also higher than that of other populations. Brenda W J H Penninx et al. reported that the increased risk of premature death of psychiatric patients might be related to the increased risk of metabolic syndrome (12). This study analyzes the pathogenesis of bipolar disorder with metabolic syndrome from the perspective of the immune system.

As is widely known, mental disorders, including BD, can influence a range of metabolic changes such as blood lipids. In our extensive literature review, we found that most studies have focused on the impact of BD on MS (13–15).

However, what piques our interest is the shared risk factors between BD and MS. We are intrigued by the possibility of a dynamic interplay between them, where each condition might influence the other in a dialectical manner. Our aim is to unravel the underlying mechanisms behind this interaction and explore the feasibility of establishing a predictive diagnostic model for early detection of BD in patients with MS.

## 2. Methodology

### 2.1. Data collection

The bipolar disorder data set GSE5388, and the metabolic syndrome data set GSE98895 were provided by the GEO database^1^ (16). The specific process is shown in the detailed data set information is shown in Table 1.

**Table 1:** Detailed data set information.

GSE series	Type	Sample size		Platform
		Bipolar disorder	Control	
GSE5388	mRNA	30	31	GPL95
GSE53987	mRNA	52	55	GPL570
GSE39653	mRNA	8	24	GPL10558
		Metabolic syndrome	Control	
GSE98895	mRNA	20	20	GPL6947

### 2.2. The identification of DEGs

The differential expression screening approach Limma (linear models for microarray data) is based on generalized linear models. Here, we do a differential analysis to derive differential expression using the R package limma (version 3.40.6). Genes that differ between the comparison group and the control group (17). The expression spectrum dataset we obtained is specifically used to perform multiple linear regression using the lmFit function, and then the eBays function is used to compute moderated t-statistics, moderated F-statistics, and log-odds of differential expression by empirical Bayes moderation of the standard errors toward a common value, and finally to determine the significance of the difference for each gene. Create filters for genes with differential expression: To find genes that are differentially expressed, use the following filters: Set a criterion of |log2 Fold Change (FC)| > 1 for bipolar disorder filtering and > 1.5 for metabolic syndrome filtering. Also, use P=0.05 as significance level. Genes with notable changes were found using these tests.

### 2.3. Weighted gene co-expression network analysis and modular gene selection

First, we calculated the MAD (Median Absolute Deviation) of each gene using the gene expression profile (18). We then deleted the top 50% of genes with the smallest MAD, and then excluded outlier genes and samples using the goodSamplesGenes function of the R package WGCNA. Specifically, you can utilize WGCNA to create a scale-free co-expression network. For all pair-wise Genes, the average linkage approach and Pearson’s correlation matrices were first used. Then, using the power function A_mn = |C_mn| (C_mn = Pearson’s correlation between Gene_m and Gene_n; A_mn = adjacency between Gene m and Gene n), a weighted adjacency matrix was created. β was a soft-thresholding parameter that may highlight significant gene-to-gene correlations and punish less significant ones. The Adjacence Was Transformed Into A Topology Overlap Matrix (Tom), Which Could Measure the Network Connectivity of a Gene DEFINED AS the Sum of I TS Adjacence with All Other Genes for Network Gene Ration, and the CorreSponding Disabilities (1-TOM) Were Calculated. After Selecting the Power of 16, The Adjacence Was Transformed Into A Topology Overlap Matrix (Tom). Average linkage hierarchical clustering was carried out using the TOM-based dissimilarity measure with a minimum size (Gene group) of 30 for the Genes dendrogram in order to arrange genes with comparable expression profiles into gene modules. The sensitivity should be set to 3; We determined the module’s eigengene dissimilarity, selected a cut line for the dendrogram, and combined certain modules in order to further investigate the module. It is important to note that after merging the modules with a distance of less than 0.25, we eventually acquired 10 co-expression modules. The gray module is regarded as a group of genes that cannot be allocated to any module, which is the most crucial aspect. Then, using truncation criteria (|MM| > 0.8), we calculated the expression correlation between the gene and the module feature vector to obtain MM. This allowed us to search for hub genes with high connectivity in clinically significant modules.

### 2.4. Gene function enrichment analysis

For gene set function enrichment analysis, KEGG rest API^2^ was used to retrieve the latest KEGG pathway gene annotation, which was used to map genes to the background set, and the ‘clusterProfiler’ R software package (19) was employed for enrichment analysis to obtain the outcomes of gene set enrichment. The minimum gene was set as 5, the maximum gene set as 5,000, and the *p* value <0.05 was taken as statistically significant.

### 2.5. PPI network construction

STRING was employed to develop the protein–protein interaction network (PPI) (20). The minimum required interaction score was set to medium confidence (0.400). PPI vector graphics and protein node degrees were downloaded directly from the website for further analysis.

### 2.6. Machine learning

In this study, ‘glmnet’ and “randomForest” R package was used to integrate survival status and time and gene expression data, the LASSO cox method and RandForest was used for regression analysis (21, 22). Moreover, five-fold cross-validation was also set to optimize the model. We set the Lambda value to 0.08.

Data integration: Integrate gene expression, survival time, and survival status data into a single dataset. To make sure that each sample is connected to its matching survival status, survival time, and gene expression values, this may entail combining and structuring the various data tables. LASSO Cox Regression: The R package ‘glmnet’ was used to do regression analysis using the LASSO Cox approach. Least Absolute Shrinkage and Selection Operator, sometimes known as LASSO, is a regression analysis technique for high-dimensional data. It has the ability to use variable selection to exclude key gene features linked to time and survival status. Random Forest Regression: 'randomForest’ is a R package that was used to do the regression analysis. Random Forest is an ensemble-based machine learning approach appropriate for both classification and regression issues. Here, it was used for regression analysis, possibly to evaluate the intricate connections between various genetic features, time, and survival status. Setting the lambda value: The lambda value in the LASSO regression is set to 0.08. In LASSO, the regularization parameter lambda is employed to regulate the model’s feature sparsity. Different numbers of gene characteristics can be chosen to create regression models by changing the Lambda value.

The ROC analysis was carried out using ‘pROC’ R software package to obtain AUC (23). The CI function of pROC was used to evaluate AUC and confidence intervals to obtain the final AUC result. And use sangerbox to visualize (24).

### 2.7. Immune infiltration analysis

The Cibersort (25) in the R software package was employed to analyze the immune cell infiltration, and the ‘corrplot’ (26) in the R software package was cited to draw the heat map of infiltrating immune cells.

## 3. Results

### 3.1. DEGs identification

**Figure 1:**
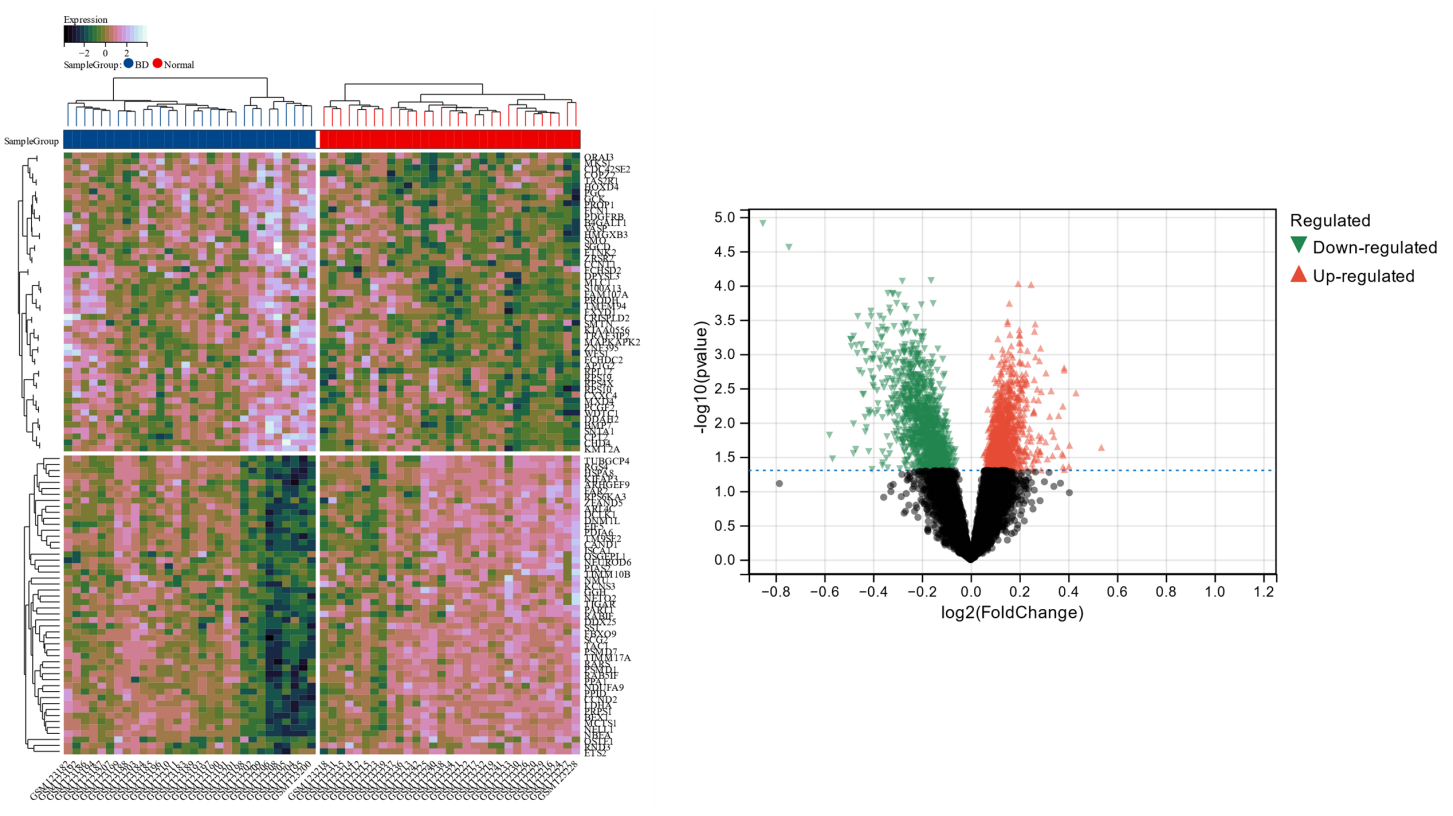
The heat and volcanic maps of DEGs identified in a bipolar affective disorder data set.

**Figure 2:**
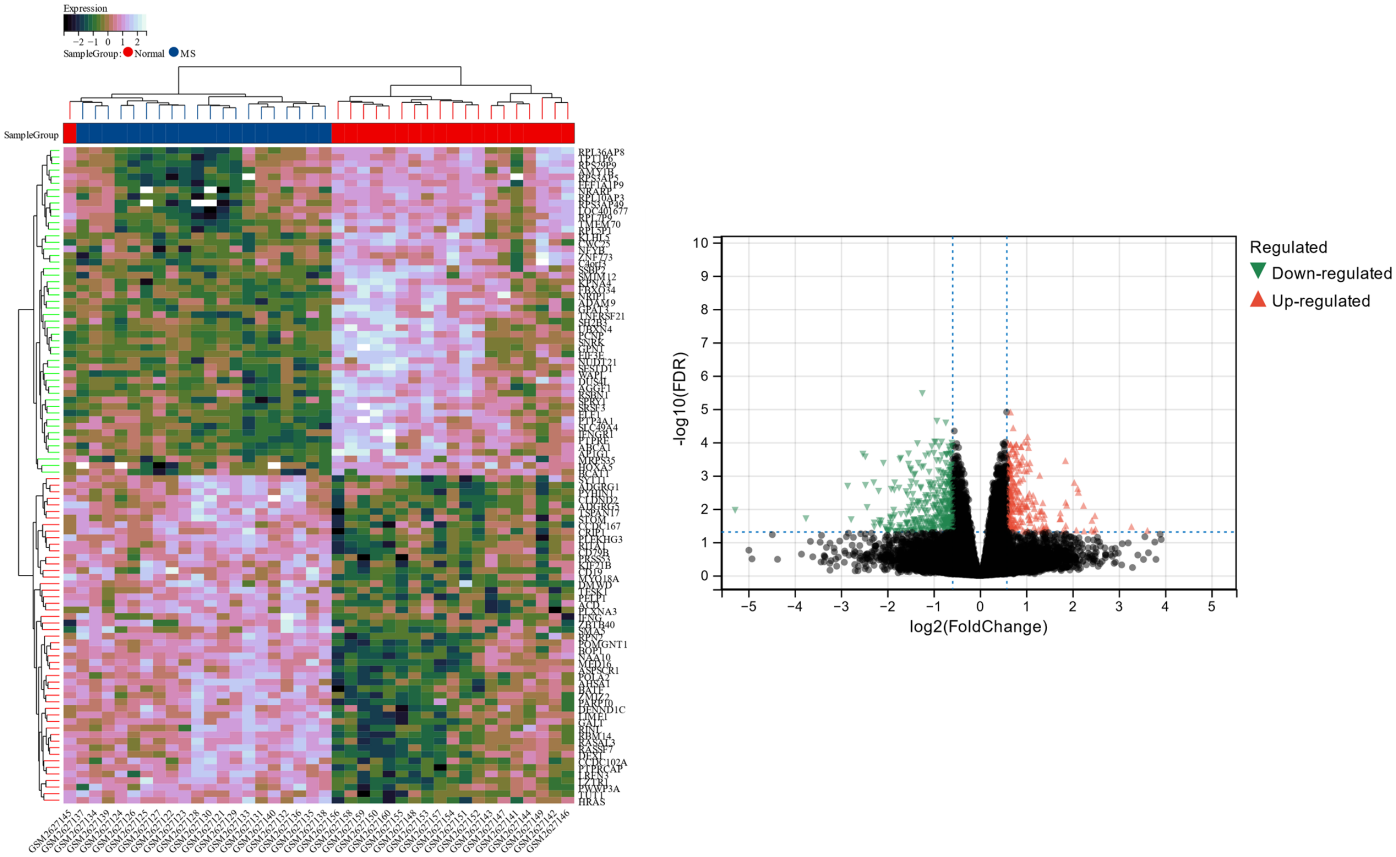
Heat map and volcano map of DEGs identified in metabolic syndrome data set.

The Limma tool was utilized to find 2,289 DEGs in the bipolar disorder data set, of which 1,096 showed up-regulation and 1,193 showed down-regulation. The heat map and volcano map of bipolar disorder DEGs are shown in Figure 1. From the metabolic syndrome data set, 579 DEGs were chosen, of which 342 showed down-regulation and 255 showed up-regulation. The heat map and volcano map of the metabolic syndrome DEGs are shown in Figure 2.

### 3.2. Weighted gene co-expression network analysis and modular gene selection

**Figure 3:**
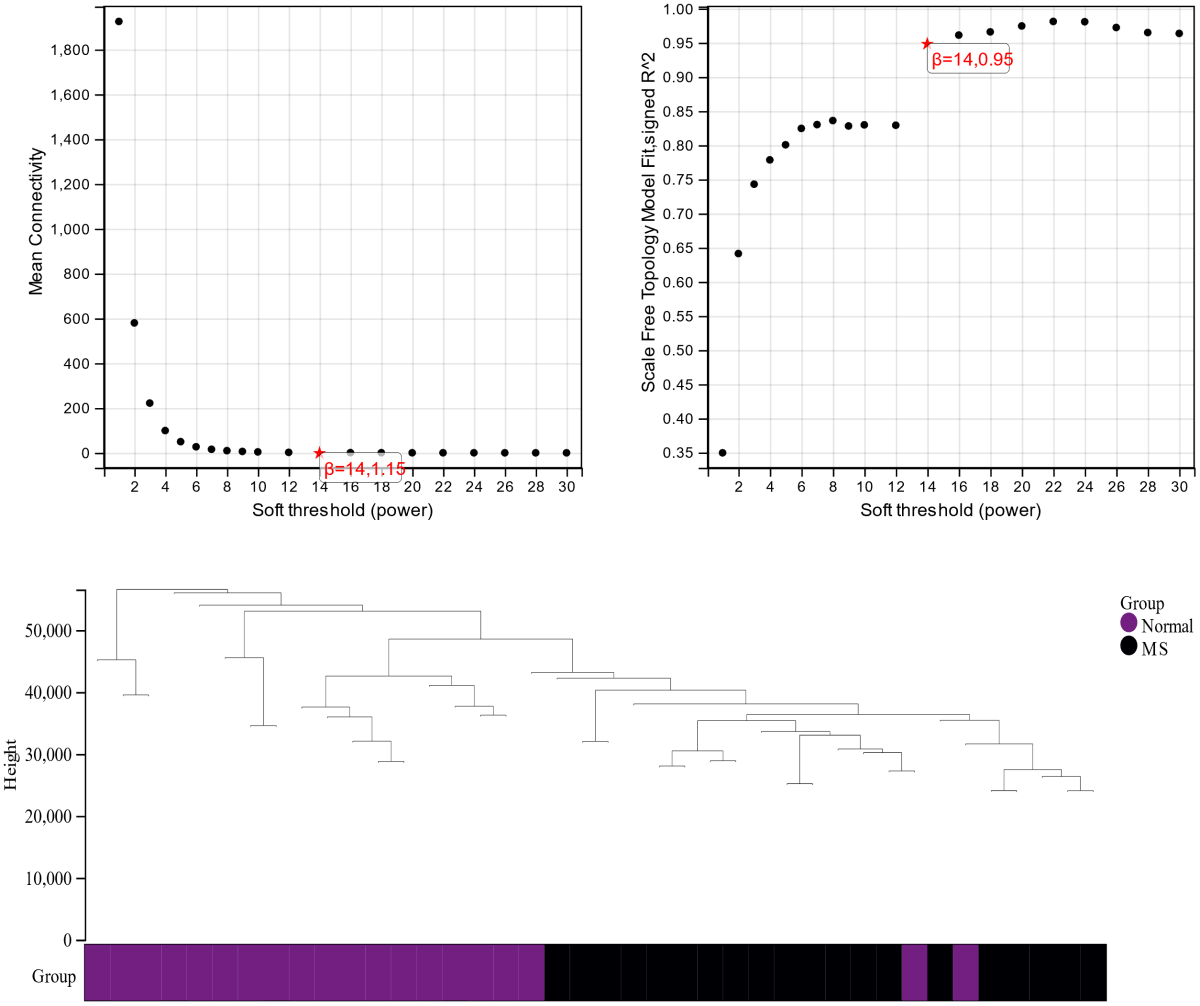
Cluster dendrogram of metabolic syndrome and control group.

**Figure 4:**
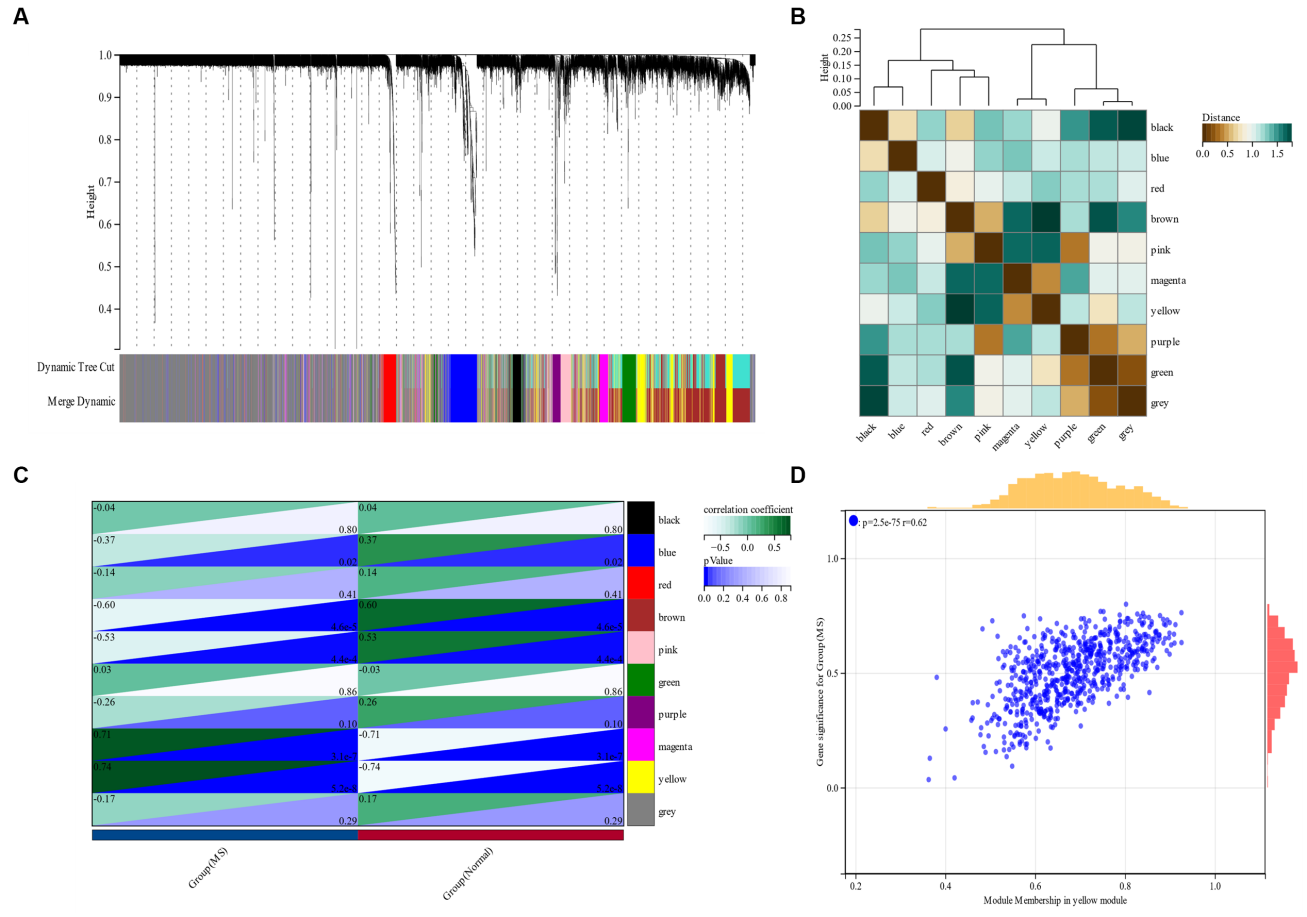
**(A,B)** Metabolic syndrome gene co-expression module (GCM). **(C)** Correlation between metabolic syndrome and GCM. **(D)** Correlation between module members and gene significance in the metabolic syndrome’s yellow module.

β is a soft threshold parameter that can highlight the high degree of gene-to-gene correlation and penalize a low degree of correlation. Here we chose β = 14 as the soft threshold for this study and drew a cluster tree of metabolic syndrome and the control group (Figure 3). On this basis, ten gene co-expression modules (GCMs) were generated (Figures 4A,B), and the correlation between metabolic syndrome and GCM was shown (Figure 4C). The results showed that there was a significant association between metabolic syndrome and the yellow module (691 genes; correlation coefficient = 0.74, *p* = 5.2 * 10–8). In the metabolic syndrome’s yellow module, we also evaluated a relationship between module members and gene significance and found a substantial positive association (*r* = 0.62; Figure 4D).

### 3.3. Functional enrichment analysis of metabolic syndrome

**Figure 5:**
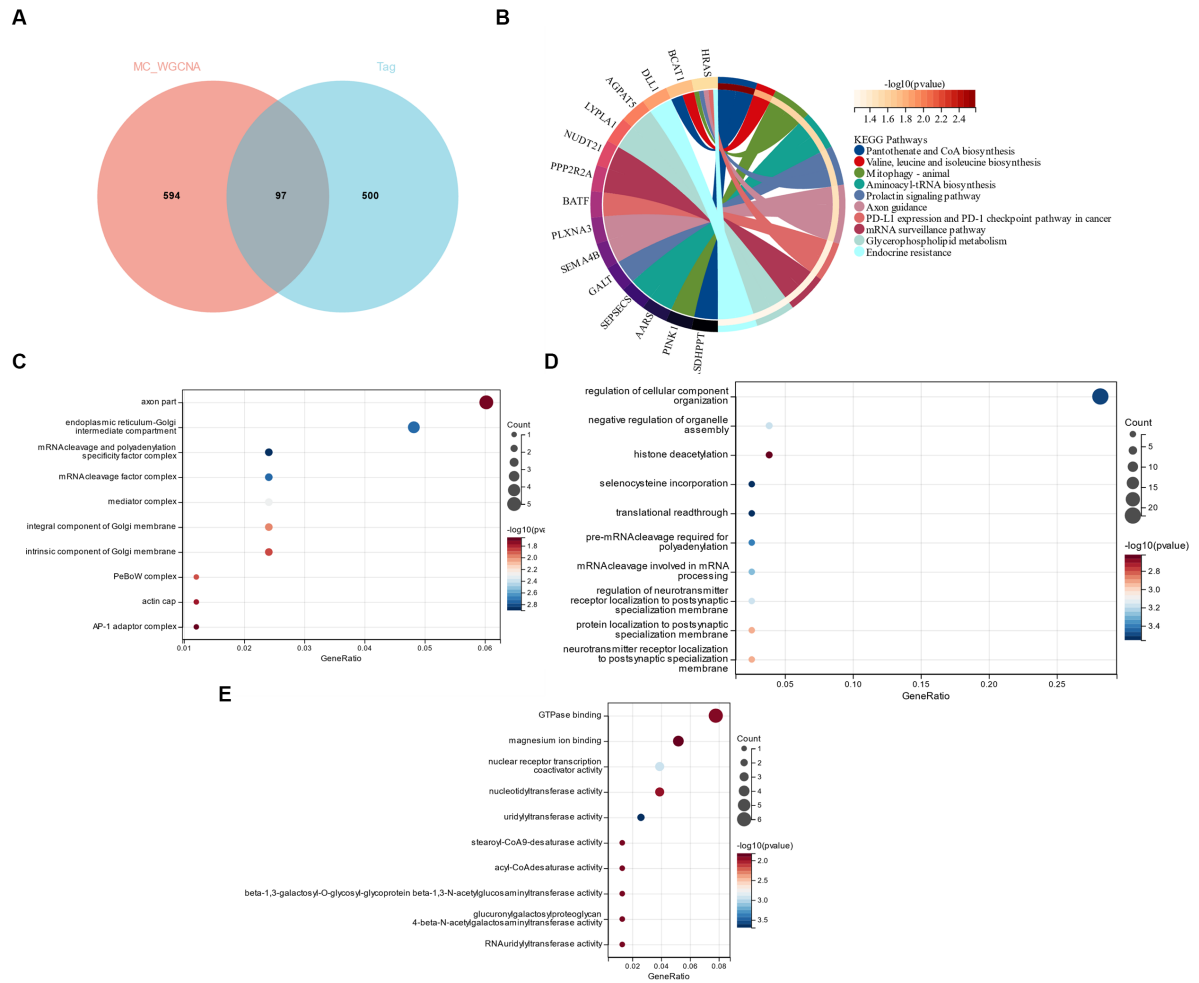
**(A)** Functional enrichment analysis of cross genes of Limma and WGCNA module genes. **(B)** KEGG analysis. **(C)** GO analysis of cell composition (CC). **(D)** GO analyzes biological processes (BP). **(E)** GO analyzes molecular function (MF).

According to the research of Zhou et al. (27) GSE98895 is a relatively new data set of metabolic syndrome; therefore, based on the cross genes of the Limma and WGCNA module genes, a functional enrichment analysis was performed. Through the intersection of 579 DEGs and 691 genes in the yellow module, 97 common genes (CGs) were selected (Figure 5A). CSs were most enriched in “Axon guidance” and “Valine, Leucine and Isoleucine Biosynthesis,” according to KEGG analysis (Figure 5B). GO analysis showed that in terms of cell components (CC), CG was mainly located in the “axon part” and “endoplasmic reticulum-Golgi intermediate compartment” (Figure 5C). The main biological processes (BP) of CGs include “regulation of cellular component organization,” “negative regulation of organelle assemble” and “histone deacetylation” (Figure 5D). Molecular function (MF) showed that the most important items in CGs were “GTPase binding” and “magnesium ion binding” (Figure 5E).

### 3.4. Enrichment analysis of bipolar disorder and metabolic syndrome and node gene identification constructed by PPI network

**Figure 6:**
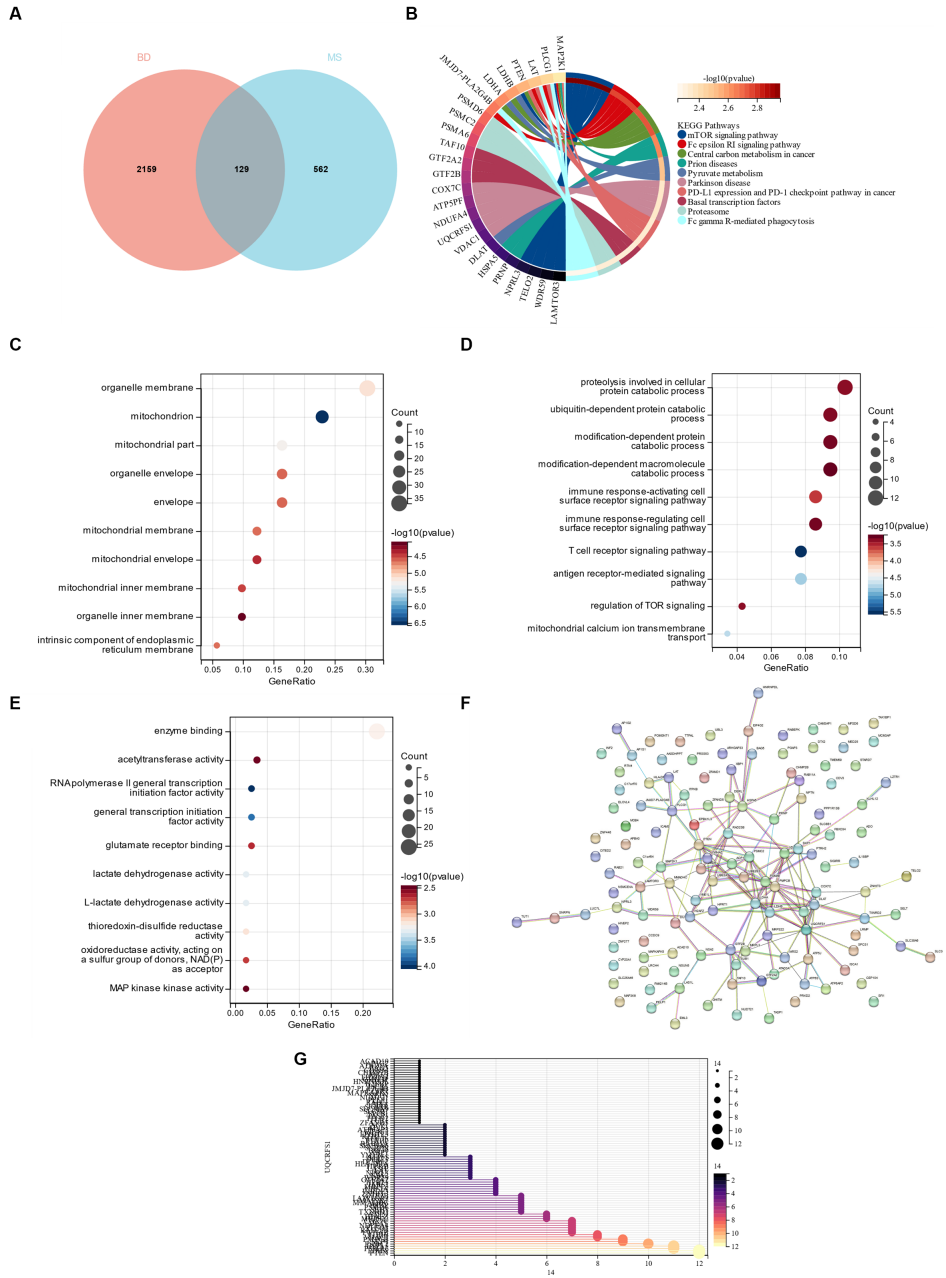
**(A)** Cross between DEG in bipolar disorder and yellow module gene in metabolic syndrome. **(B)** KEGG analysis of cross genes. **(C)** GO analysis of cell composition (CC). **(D)** GO analyzes BP. **(E)** GO analysis MF. **(F)** PPI network interaction diagram. **(G)** 86 gene nodes in PPI network.

A total of 129 genes were obtained by crossing the yellow module genes in the DEGs of bipolar disorder and metabolic syndrome (Figure 6A). These genes were primarily enriched in the “mTOR signaling pathway” and “Parkinson’s disease,” according to the KEGG enrichment analysis (Figure 6B). According to GO analysis, CG was mostly found in “organelle membrane” and “mitochondrion” in terms of cell components (CC; Figure 6C). The main biological processes (BP) of CGs include “proteolysis taking part in cellular protein catabolic process,” “ubiquitin-dependent protein catabolic process,” “modification-dependent protein catabolic process” and “modification-dependent macromolecule catabolic process” (Figure 6D). Molecular function (MF) showed that the most important item in CGs was “enzyme binding” (Figure 6E). A PPI network was constructed, and the analysis showed that 86 genes and the PPI network could interact, and the results were sorted according to the number of nodes (Figures 6F,G).

### 3.5. Identifying candidate genes through machine learning

**Figure 7:**
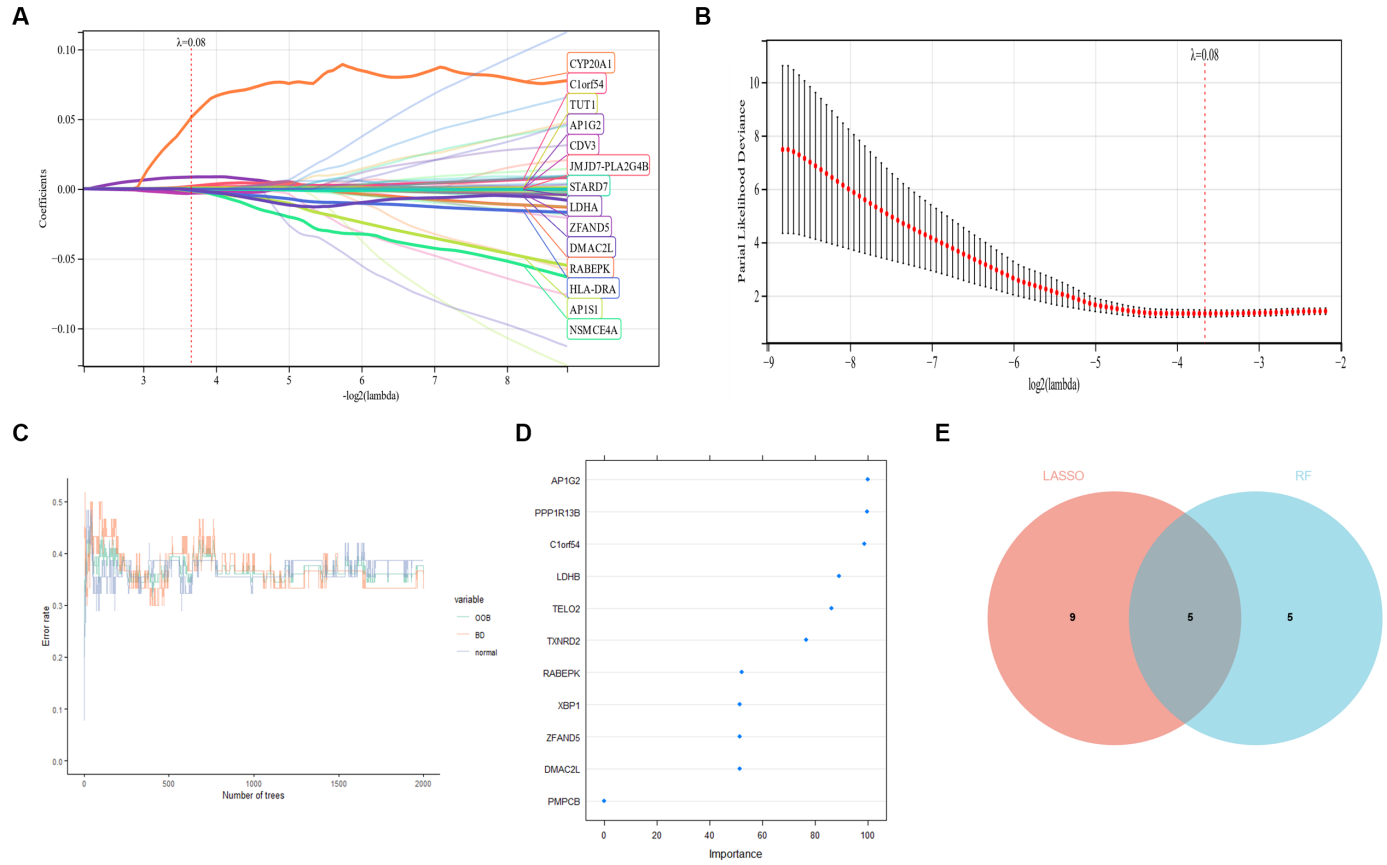
**(A,B)** LASSO regression candidate gene identifying. **(C,D)** Applied RF regression for candidate gene screening. **(E)** Five candidate diagnostic genes are identified via the above two algorithms.

LASSO regression was used to identify candidate genes, and as per the findings, 14 potential candidate biomarkers (Figures 7A,B) were identified. We also applied RF regression for candidate gene screening, from which ten potential candidate biomarkers were revealed (Figures 7C,D). The results of the two machine learning screens were then intersected and analysed, resulting in five candidate genes (AP1G2, C1orf54, DMAC2L, RABEPK, ZFAND5; Figure 7E), and subsequent diagnostic value evaluation has been carried out.

**Figure 8:**
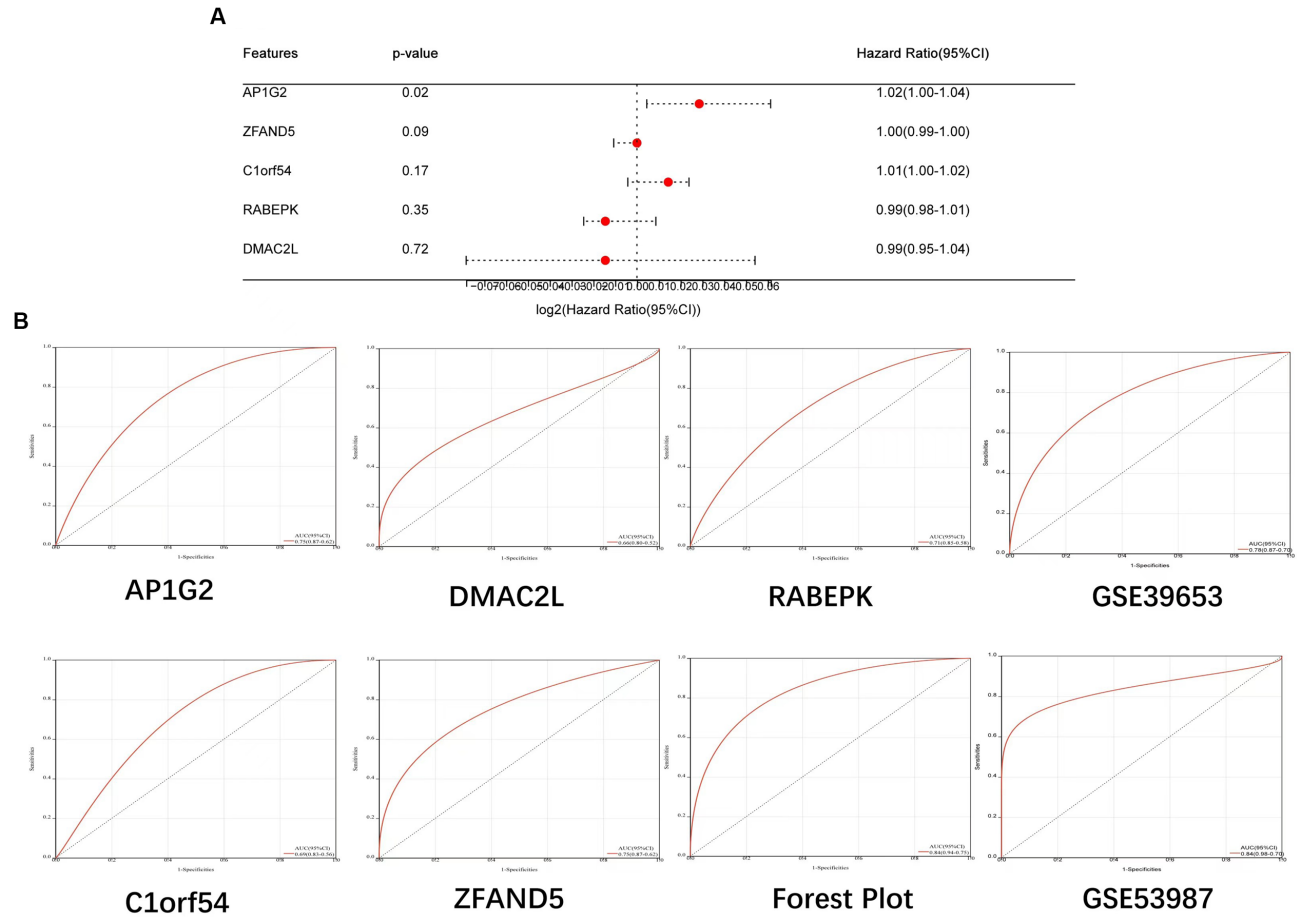
**(A)** Construction of Forest Plot based on five candidate central genes. **(B)** ROC curves of 5 potential candidate genes, Forest Plot and Validation Team were established.

Construction of Forest Plot based on five candidate central genes (Figure 8A). ROC curves of 5 potential candidate genes and Forest Plot were established, and the AUC and 95% CI of each item were calculated to assess their specificity and sensitivity. AP1G2 (AUC = 0.75, CI = 0.87–0.62), C1orf54 (AUC = 0.69, CI = 0.83–0.56), DMAC2L (AUC = 0.66, CI = 0.80–0.52), RABEPK (AUC = 0.71, CI = 0.85–0.58), ZFAND5 (AUC = 0.75, CI = 0.87–0.62) and Forest Plot (AUC = 0.84, CI = 0.94–0.75; Figure 8B). The results showed that all candidate genes had high diagnostic value for bipolar disorder with metabolic syndrome.

Further, we validated the diagnostic model on two independent bd datasets, gse39653 (AUC 0.78,CI 0.87–0.70) for the pbmc sample and gse53987 (AUC 0.84,CI 0.98–0.70) for the brain tissue sample (Figure 8B). The results showed that the model also achieved a high diagnostic value in the revalidation group.

### 3.6. Immune cell infiltration analysis

**Figure 9:**
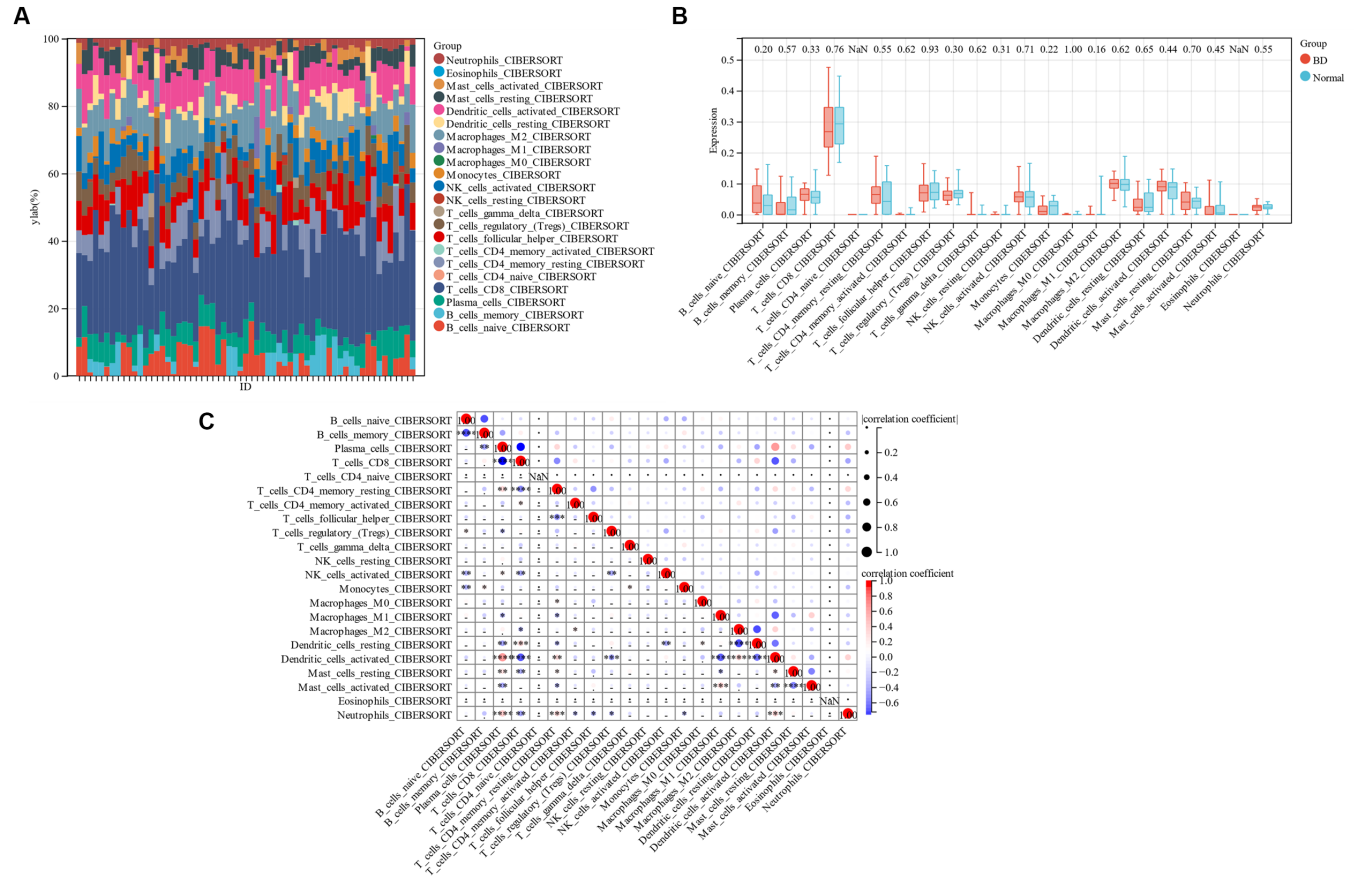
**(A)** Bar chart of 22 kinds of immune cells. **(B)** Comparison of 22 types of immune cells between patients with bipolar disorder and control group. **(C)** 22 types of immune cells.

To compare the proportion of each sample’s 22 different types of immune cells between the bipolar disorder group and the control group, a bar chart was constructed (Figure 9A). Figure 9B shows that while the numbers of memory B cells, CD4 resting T cells, monocytes, and resting dendritic cells are lower in individuals with bipolar disorder, the levels of naïve B cells, plasma cells, and resting mast cells are greater. There is a differential infiltration correlation according to the correlation of 22 types of immune cells, excluding naïve T cells and Eosinophils (Figure 9C).

## 4. Discussion

More than 1% of people worldwide suffer from bipolar disorder, and a meta-analysis by Davy Vancampfort et al. found that 32.6% of individuals with severe mental illness also have concomitant metabolic syndrome (28, 29). Patients with bipolar disorder are more likely to have metabolic syndrome, which can negatively affect executive functioning (30). The co-morbid mechanisms of bipolar disorder and metabolic syndrome have not yet been thoroughly studied; thus, this study employed a machine learning approach to identify a link between the two.

This study examined five genes with a potential link to bipolar illness, none of which has been linked to previous research on the condition. AP1G2 (Adaptor Related Protein Complex 1 Subunit Gamma 2) is a Protein Coding gene. Diseases associated with AP1G2 include Tracheoesophageal Fistula With Or Without Esophageal Atresia and Esophageal Atresia. Among its related pathways are trans-Golgi Network Vesicle Budding and Vesicle-mediated transport. Gene Ontology (GO) annotations related to this gene include binding and obsolete protein transporter activity (31). Additionally, AP1G2 is a linked gene in both Developmental and Epileptic Encephalopathy (32) and Intellectual Developmental Disorder, Autosomal Dominant (33). These two conditions share certain similarities with bipolar disorder, supporting the significance of our findings.

A gene for protein-coding is called RABEPK (Rab9 Effector Protein with Kelch Motifs). Retrograde transport at the Trans-Golgi Network and vesicle-mediated transport are two of its associated processes. Transport is mediated by vesicles and the network (28). We did not search for disorders linked to RABEPK, however Rachel L. Kember’s study This gene was discovered to be linked to opioid use disorder (OUD) (34), and previous research has shown a link between OUD and BD (35). Additionally, our candidate gene is correspondingly validated by this.

The proteins encoded by both AP1G2 and RABEPK are associated with the vesicular pathway, which has been a hot topic of research for several years, and both have shown opposite risk for the development of bd in our study. We are conducting further analysis of this pathway in recent studies, which we believe will help to unravel the mechanisms underlying the prevalence of psychiatric disorders, including bd.

ZFAND5 is a less studied mrna that has been associated with lipoprotein metabolism. zfand5 enhances the stability of ARE-RNA by competing for mRNA binding with TTP, a transcript associated with inflammatory mediators. This is consistent with our analysis in immune infiltration.

With advances in molecular genetic phenotyping studies, the links between social behavior and genetic variation to protein and receptor function and disease development are increasingly being revealed. The findings of this study shed light on the possibility that metabolic disorders, as an exposure factor, may cause alterations in the genetic markers of qtl, which in turn lead to differential expression of mrna transcripts, which in turn play a role in translational protein function (including alterations in vesicular transport pathways and other metabolic pathways as well as in the immune microenvironment) and finally lead to the outcome of bd. We think this is a very interesting and interesting line of research, and for some time now our team has been working on a Mendelian randomization study analysing neurodegenerative diseases and social behavior. Although this study has not yet fully elucidated the cause-effect relationship, we believe that this step in the analysis of the mrna and protein levels is important and meaningful.

In addition, the diagnostic target genes and risk model formulas obtained from this study will be useful for early diagnosis and individualized risk indication for patients suspected of having bd in the metabolic syndrome, and our subsequent studies on genetic markers for qtl will be useful for earlier risk assessment of bd.

## 5. Conclusion

A total of 5 candidate genes were identified, and all of them had diagnostic value, providing potential candidate genes for bipolar disorder with metabolic syndrome.

## Data availability statement

Publicly available datasets were analyzed in this study. This data can be found at: https://www.ncbi.nlm.nih.gov/geo/ with accession numbers GSE5388 and GSE98895.

## Author contributions

YF and JS wrote the main manuscript text. ML, JH, and HY provide experimental help and all authors reviewed the manuscript. All authors contributed to the article and approved the submitted version.

## Footnotes

^1^ https://www.ncbi.nlm.nih.gov/geo/

^2^ https://www.kegg.jp/kegg/rest/keggapi.html

## Glossary

BD: Bipolar disorder
MS: Metabolic syndrome
GEO: Gene Expression Omnibus
DEGs: Differentially expressed gene
WGCNA: Weighted gene co-expression network analysis
PPI: Protein–protein interaction
LASSO: Least absolute shrinkage and selection operator
RF: Random Forest
ROC: Receiver operating characteristic
MAD: Medium Absolute Development
GCMs: Co-expression modules
GO: Gene Ontology
OUD: Opioid use disorder
CC: Cell components
BP: Biological processes
MF: Molecular function
